# “When you provide abortion services, you are looked upon as a bad guy”: experiences of abortion stigma by health providers in Nigeria

**DOI:** 10.1080/16549716.2024.2401849

**Published:** 2024-10-30

**Authors:** Friday Okonofua, Lorretta Ntoimo, Louise Bury, Suzanna Bright, Lesley Hoggart

**Affiliations:** a African Centre of Excellence in Reproductive Health Innovation, University of Benin, Benin, Nigeria; b Women’s Health and Action Research Centre, Benin City, Nigeria; c Department of Demography and Social Statistics, Federal University Oye-Ekiti, Oye-Ekiti, Nigeria; d Independent Consultant, Colchester, UK; e Royal College of Obstetricians and Gynaecologists, London, UK; f Faculty of Wellbeing and Language Studies, The Open University, Milton Keynes, UK

**Keywords:** abortion, stigma, health providers, reproductive health, Nigeria

## Abstract

**Background:**

Abortion stigma as reported globally has been inadequately documented empirically in Nigeria, Africa’s most populous country with a restrictive abortion law and a high rate of unsafe abortions.

**Objective:**

The objectives of this study were to investigate the ways in which abortion stigma is experienced by Nigerian health professionals and how such experiences influence health professionals’ practice of safe abortion and post-abortion care.

**Methods:**

The study utilized qualitative research consisting of in-depth interviews with 10 abortion providers. We elicited information with an open-ended interview guide that investigated the understanding of participants’ experiences of abortion stigma in Nigeria. The data were analysed qualitatively and thematically using Atlas.ti.

**Results:**

The themes centred on perceptions and experiences of stigma among the providers interviewed. Participants’ experiences of abortion stigma included the following: being treated differently to other health professionals; experiencing disapproval and disrespect; name-calling and societal judgement; tagging and profiling of clinics by anti-abortionists; and social isolation. Participants attributed stigma to cultural and religious beliefs, the restrictive national abortion law, and pointed to hypocrisy. Some reported effects of stigma on providers included a feeling of insecurity, social exclusion, secrecy, and insincerity in clinical practice, discouragement, and guilt feelings. Despite the negative impacts, many respondents reported a sense of satisfaction stemming from their views that they were saving lives.

**Conclusion:**

Systematic efforts to address these adverse factors could reduce the level of stigma experienced by providers, with a potential follow-through effect of improving women’s access to safe abortion care in Nigeria.

## Paper context

- **Main findings:** Healthcare providers in Nigeria perceive abortion stigma which they feel is driven by cultural and religious beliefs and restrictive national laws.
- **Added knowledge:** Nigerian healthcare professionals working in abortion care perceive stigma as being treated differently from other health professionals; disapproved and disrespected, and judged by society. Despite this, providers can experience satisfaction from knowing their work saves lives.
- **Global health impact for policy and action:** Greater attention to the impact of abortion stigma on healthcare providers in Nigeria is required to ensure the workforce is supported and women can access care.

## Background

Induced abortion is currently one of the most challenging issues relating to public health and the discourse on sexual and reproductive health and rights in Nigeria. Estimated 1.25 million abortions (33 per 1000 women aged 15–49 years) take place in Nigeria each year, resulting in about 3000 deaths and 212,000 severe complications [1]. These high rates of morbidity and mortality associated with abortion have been attributed to a restrictive law that limits the use of abortion to cases when the life of the woman is threatened [2]. In these cases, doctors trained to provide abortion care in registered hospitals can use medical or surgical methods for abortions following clear guidelines. Medical methods involve using mifepristone and/or misoprostol for pregnancies of 0 to 24 weeks, while surgical methods include Manual Vacuum Aspiration (MVA) for pregnancies under 12 weeks and Dilatation and Evacuation (D&E) for pregnancies of 12–14 weeks. Post-abortion checks and care are mandatory after each procedure. Over 70% of the induced abortions in Nigeria are unsafe and carried out clandestinely by health professionals and para-medical professionals who are not trained to provide abortion care [1]. Although Nigeria’s Federal Ministry of Health has published guidelines permitting termination of pregnancies under the law [3], only a few health practitioners are willing to carry out induced abortions when compared to other reproductive health services [4,5]. Some reasons that have been proffered for the law practice of safe abortion care by health professionals in Nigeria include limited knowledge of the abortion law; perceptions that all abortions are prohibited in the country; religious and moral concerns; and the fear of legal and social retribution [4–8]. These underpin abortion stigma.

The fear of stigmatisation has been reported as a barrier that constrains the provision of safe abortion care by health professionals in many low- and middle-income countries with restrictive abortion laws [9]. Abortion stigma has been defined as ‘a shared understanding that abortion is morally wrong, and/or socially unacceptable’ [10]. While most studies on abortion stigma have focused on women requesting or experiencing induced abortion [11,12], only a few studies have examined the full ramifications of the experiences of abortion stigma among health professionals. Several reviews of abortion stigma have reported that providers often report stigma as undermining their work on abortion care, with many recounting experiences of devaluing remarks, including social and physical harassment [12,13].

In Nigeria, whilst some research has reported women’s experiences and perceptions about abortion and their fears related to it [14,15], there is a dearth of literature on the experiences of abortion stigma among health professionals. Given that Nigeria is a country with documented evidence of being occupied in the moral, ethical, and religious debate relating to abortion [16–18], it is highly probable that health professionals will be impacted by these discourses resulting in a high likelihood of both ‘felt’ and ‘enacted’ stigma among health practitioners. Felt stigma refers to an expectation of discrimination whilst enacted stigma refers to experiences of discrimination. We designed this qualitative study to investigate the ways in which abortion stigma is experienced among Nigerian health professionals, and how such experiences may impact their use and practice of safe abortion care (AC) and post-abortion care (PAC). Globally, safe abortion care has come to be recognized as a human right and gender-based issue for which women deserve to be afforded the rights to responsible and independent decision-making and choice [19]. Our key research questions were as follows: how, and to what extent, do health professionals who provide safe abortion services in Nigeria experience abortion stigma in their work? And how may these experiences affect their practice of safe abortion? We believe the results of the study will be useful for demonstrating the drivers and impact of abortion stigma on providers, identifying solutions, and ultimately supporting healthcare providers to offer care, thus increasing the availability of safe abortion and reducing morbidity and mortality.

## Methods

The study was part of the Making Abortion Safe (MAS) advocacy programme, implemented in five African countries – Nigeria, Rwanda, Sierra Leone, Sudan, and Zimbabwe – by sexual and reproductive health and rights (SRHR) Champions selected by the Royal College of Obstetricians and Gynaecologists (RCOG). The project aimed to improve women’s and girls’ access to high quality, safe abortion services, post-abortion care, and/or post-abortion contraception by addressing specific barriers to care in these countries. The study used a mixed methods approach to investigate different aspects of abortion providers’ experiences of abortion-related stigma through two linked phases:

- Phase 1 consisted of a global online cross-sectional survey of abortion care providers
- Phase 2 consisted of in-depth interviews among selected AC/PAC providers conducted in the five MAS programme focus countries: Nigeria, Rwanda, Sierra Leone, Sudan, and Zimbabwe.

This paper focuses on the interviews conducted in Nigeria under Phase 2. The purpose of the interviews was to provide a more detailed and nuanced understanding of the manifestations of stigma experienced by different types of providers of abortion care in different settings and to explore how experiences of stigma impact their work and personal lives.

### Study sample and recruitment

The study comprised in-depth interviews with a range of AC/PAC providers who were purposively selected across six geopolitical zones of Nigeria (see Table 1). Administratively, Nigeria is divided into 36 states and the federal capital territory, Abuja. The 36 states and Abuja are further categorized into six geo-political zones, also called regions: North central, North east, North west, South east, South-south, and South west. States in the same geographical area with cognate cultures are grouped together into a geo-political zone. The sample included doctors, nurses and midwives who were trained to provide AC and PAC services across different sectors: private, public, and non-governmental organisations (NGO). The sample represented differences in types of clinics by locations, experiences of providers, volumes of patients seen, and types of ownership (public, private, and NGOs)

**Table 1.** Profile of the respondents.

Type of provider	Sex	State/Location	Type of facility	Duration of work in AC/PAC
Nurse/Midwife	Female	Abuja	NGO clinic	5 years
Nurse/Midwife	Female	Abuja	Private hospital	15 years
Medical Doctor	Male	Gombe	Public hospital	23 years
Medical Doctor	Male	Jigawa	Public hospital	20 years
Medical Doctor	Male	Gombe	Private hospital	>20 years
Medical doctor	Male	Abia	Private hospital	20 years
Medical Doctor	Male	Abia	Private hospital	21 years
Nurse	Female	Oyo	Public hospital	4 years
Nurse	Female	Edo	NGO clinic	2–3 years
Nurse/midwife	Female	Oyo	Private clinic	>25 years

Eligible participants for the interviews were identified by in-country SRHR Champions,^1^^1^SRHR Champions were public health and clinical professionals working on a voluntary basis with the MAS programme to support national level advocacy work to increase access to quality abortion care. in consultation with the Principal Investigator (LB), through their known networks of international SRHR organisations, agencies working in Nigeria, and public/government institutions.

The inclusion criteria for participation in the interviews were as follows:

- aged ≥18
- works in Nigeria
- is a provider (trained clinician) of safe abortion care or post-abortion care within the legal framework of the country
- understands English or the national language,
- has access to a mobile phone or computer for a virtual/online interview, and
- consents to participate

A local researcher^2^^2^In Nigeria, an independent consultant was hired. personally contacted the identified individuals via email to invite them to participate in the study. If they agreed to participate, the interviewer sent them a copy of the study information sheet and a link to an online consent form and confirmed a time and date for the interview. Thirteen providers were invited and all accepted following confirmation of informed consent.

### Data collection

#### In-depth interview guides

The research team wrote a semi-structured topic guide to inform the interviews. The guide had a similar focus to the online survey questionnaire from Phase 1 of the study (Appendix 1) and underwent minor adaptations, in consultation with the country's SRHR Champions, to make it appropriate to the Nigerian country context.

#### In-depth interviews

Once informed consent was confirmed using the online platform, interviews were conducted by an experienced female local researcher and a medical doctor who had no prior relationship with the participants and was not part of the study. Interviews were undertaken in English and audio-recorded. All transcriptions were completed by the local researcher. No interviews were conducted face-to-face due to COVID-19 restrictions. The local researcher attended an online training session with the co-principal investigators to ensure quality and consistency in their interviewing technique. Interviews lasted, on average, 38 minutes. There were no repeat interviews. The interviews continued until data saturation was reached, which was judged to be after 10 interviews.

### Data processing and analysis

All interviews were recorded, transcribed, anonymized, and stored securely. All the interview data will be kept for a maximum of 5 years/a minimum of 2 years after the end of the MAS programme.

Transcripts from each interview were uploaded onto the qualitative data analysis software Atlas.ti version 6.2.25. The data analysis followed the six-step conventional approach to thematic analysis [20]. We developed a coding frame based on the themes in the interview topic guide, and other codes identified in the data using an inductive approach. The data were then coded to these codes by LN. Network views of the relevant codes are included as Appendix 2. Codes were evaluated, and overlapping codes were further classified. Subsequently, themes were produced from categories that reflected the various experiences of abortion stigma by the providers and the impacts. The manuscript is presented using the consolidated criteria for reporting qualitative research (COREQ) guideline.

## Ethical approval

Fully informed and signed consent was obtained from the participants. Ethical approval for this study was obtained from the Open University, UK (HREC/3994/Hoggart) and the National Health Research Ethics Committee of Nigeria (NHREC) on 21 October 2021, number NHREC/01/01/2007-28/10/2021.

## Results

Our analysis of the data shows how participants were aware of different constituent elements of abortion stigma, were able to identify the result of this stigma in their own life experiences, and were also able to identify the effects of these experiences on their professional practice and personal lives. This section begins by analysing the participants’ understandings of the structural underpinnings of abortion stigma. We then go on to explore participants’ experiences of abortion stigma, finally turning to the impact of these experiences.

### Structural abortion stigma in Nigeria

Participants were aware that their experiences of stigma were underpinned by structural features of Nigerian society. They spoke of the importance of religion, cultural values, and the punitive legal system. As noted earlier, the law limits the use of abortion to cases when the life of a woman is threatened. The participants had knowledge of the law on AC and PAC law in Nigeria in detail. Participants spoke of how the restrictive legal framework limited their practice and also pointed out how important it was for providers to understand the provisions:

Every healthcare worker must know what the law stipulates so that we will not fall into litigation or have cases with the police. Because our law enforcement system, really comes down very heavily when cases of criminal abortion are brought to them. So, every medical person must be very conversant with what the law says and what the law allows, and if anything like that is done, you must have the proper legal backing and proper documentation, so that you will not enter into any trouble. I have had many of my staff who had issues with the law, you know, concerning abortion. Many of them were actually not therapeutic so they had a run-in with the law, and it was not funny at all. The Nigerian legal system, especially the police, comes down very hard on medical practitioners who don’t have the proper backing or don’t have the proper indications, or don’t have the proper documentation on why they did the procedure, so that is why we must be very conversant with the laws. (Interview 3, medical doctor, public)

This participant has made it clear that acting outside the legal framework has consequences for health care professionals, but in doing so they are also indicating that therapeutic abortion can be legal with the ‘proper indications’. This is a clear sign of a structural stigma whereby abortion is illegal and therefore ‘wrong’ except in very limited life-saving circumstances.

The research participants also noted that there was also an interpretation of the restrictive law to mean that abortion is completely illegal in Nigeria, thus reinforcing the point made by this medical doctor.

Participants also explained that in Nigeria stigma is an age-long attitude to the termination of pregnancy that is rooted in religious and cultural beliefs about life and procreation.

I think it is because of our cultural values the society we find ourselves, and the belief system they believe that okay … if you terminate a pregnancy that you don’t want, you are killing. (Interview 1, nurse/midwife, NGO)

Because of what some religious leaders talk about, they say you are trying to stop procreation, so why must you do family planning, whereby God has asked that you should go into the world and multiply. That’s some people’s belief. (Interview 7, medical doctor, private)

Some participants, however, spoke of an evident hypocrisy whereby religious and cultural values are invoked in public, but in private ‘highly placed people’ may behave very differently.

Because it is hypocrisy. They judge because they are hypocrites, because actually all these people who judge, do these things. They do it privately, but because it doesn’t come out to the open, they will have that kind of effrontery to judge people who are doing it wholeheartedly. But even talking about religion, churches, and even the congress people, most highly placed people, do all these things [abortion] discretely. But people who do it openly, they condemn them. The reason for this is hypocrisy… (Interview 2, nurse/midwife, private)

These views show an understanding of the structural underpinnings of abortion stigma, in particular the legal framework, and cultural and religious beliefs. The third quotation, with a focus on hypocrisy, shows the strength of this framing as ‘highly placed people’ seek abortion services/do it ‘privately’.

### Experiences of abortion stigma

In this section, we present participants’ narratives of their experiences of abortion stigma – enacted stigma. It was rare for participants to deny experiences of stigma though there was some evidence of this:

I: Have you experienced any stigmatizing?

R: Not to my awareness, you know I cannot vouch for maybe in my absence, but not to my awareness. (Interview 2, nurse/midwife, private)

Most participants spoke of having experienced abortion stigma in various forms and with different intensities. Their experiences of stigma covered different dimensions, including disapproval and disrespect; name-calling and societal judgement; and the negative profiling of clinicians and providers as abortionists. We were also able to distinguish differential experiences depending on participants’ specific areas of work and gender.

One way in which disapproval and disrespect primarily manifested was through AC and PAC providers being treated differently from other reproductive health providers. In particular, participants felt that AC and PAC providers were being stigmatized by fellow health workers.

Yes, just like I said that when you provide these abortion services, you are looked upon as a bad guy, the person is looked upon as not very good. (Interview 5, medical doctor, private)

It does happen with non-health colleagues in the work area. When a client walks in at the reception they will, ‘she is for you, I don’t have any business with her.’ So, without the client telling them what they come for, they will conclude that the people walking in are actually for me. So, even if I am eating or I am doing some recordings, they will call my attention. It seems these people walking in are for you, because from their look of face, it is for you. (Interview 9, nurse, NGO)

The sense of disapproval could be attributed to the view that abortion work was outside what would be expected of a nurse.

The title ‘post-abortion care’ is not in the schedule of duty of a nurse. So, for me carrying out that work I face disapproval among my colleagues, and disrespect. (Interview 8, nurse, public)

Such negativity was also evident outside work with AC and PAC providers describing loss of friends and social isolation when the details of their work became known.

… so there is this stigma in the system social wise, people knowing that this is what you are doing, socially, there is a stigma, this is what we are doing, you are helping to terminate babies and life. Once it has that definition abortion, some people would just try to discriminate from you and stigmatize, condemn what you do totally because you are just doing abortions … socially, your friends. Some family member hearing what you do, would also discriminate themselves from you, and tell themselves ‘I think this person is killing’ so, you know that sense of reasoning there is always a barrier, there is always a barrier by as I mentioned now. (Interview 9, nurse, NGO)

Other forms of stigma described by the respondents include subtle and indirect comments, particularly within their communities, about work, and distancing from administrative and client relation services that have to do with AC and PAC.

Yeah, in my place it is not that common, but it happens, usually it is within the community, you have side talks, you know it is always there. (Interview 5, medical doctor, private)

Alongside disapproval, participants also experienced name-calling, and – as many of these quotes show – being labelled as ‘killers’.

Abortion care and post-abortion care providers are called abortion nurse or doctor. They are reminded by colleagues, and friends that they are killing and it is a sin. (Interview 8, nurse, public)

Yes, they see it as a sin, they call it a sin, that it is against God and man, that you are terminating a life, that you are killing a child. (Interview 8, nurse, public)

Along the same lines, there is the accusation of using ‘blood money’ to pay their tithe to the church (tithe is a percentage of salary paid by Church members to the Church), a charge very similar to the ‘killer’ accusation, which had clearly affected them negatively.

Sometimes, when we attend to these clients most of your colleagues would have something in mind. I remember what a colleague asked me: ‘at the end of the month you receive salary, do you pay tithe?’ I said ‘yes’. The person replied by saying that I was using blood money (from abortion) to pay tithe. I was really touched by the statement, which left me with severe emotional feelings. …. (Interview 9, nurse, NGO)

This invocation of the church by those labelling and name-calling illustrates the religious underpinnings of abortion stigma discussed in the previous section. Participants were also told that God would be judging them. In these circumstances, it is easy to see how abortion providers would not only expect to be treated badly but would actually experience discrimination, simply due to the nature of their work.

One of these days, I was sharing with my colleague the work I do on abortion in the NGO where I work. She said what? You mean you are an abortion nurse? I hope you don’t conduct induced abortion – if you do, you are going to hell, God is going to judge you, in fact, if you do that, you will stop being my friend. I just let her be, I couldn’t talk further. (Interview 1, nurse/midwife, NGO)

One specific way in which name-calling was experienced was the tagging and profiling of a clinic that had a range of services such as an ‘abortion centre’, and providers such as abortionists. The assumption here is that these are terms of abuse in and of themselves.

When I opened my facility in 2004, they referred it to as an abortion centre [*short sharp laugh*] so most of them who want to conduct antenatal service would prefer going elsewhere to get such services than coming to my place. They will say this guy does abortion, [and] that if they come to his clinic, maybe something will happen to their baby …. (Interview 7, medical doctor, private)

Our analysis was also able to identify different patterns associated with the experience of abortion stigma. Our participants felt that AC providers are treated differently from PAC providers. This is connected to their experiences of name-calling and labelling as ‘killers’. Whereas those who provide abortion care are seen as ‘killers’, post-abortion care providers can be viewed as ‘lifesavers’.

Of course, always there will be a difference because the abortion care is the real service where you carry out the termination of the pregnancy. When you are doing post abortion care, it is seen as if you are helping the patient to survive. So, the blame on workers doing post abortion care is less. Like one patient I saw, she tried to terminate her pregnancy. She came to the hospital bleeding profusely there was the product of pregnancy in her cervix. We completed the removal and got her settled; we transfused her with blood and gave her antibiotics. We were seen as heroes in that particular situation; meanwhile, the lady was seen as the villain and the bad person who tried to terminate her pregnancy. I was hailed and praised for helping the lady. (Interview 3, medical doctor, public)

I don’t go out willfully to start the process of an abortion, but I do PAC. So, in that situation, the stigma lessens. But for those other staff, who go out willfully, the stigma is great even in the hospital, their homes, and even in the community, the stigma is very high. (Interview 2, nurse/midwife, private)

Although most providers offer both services because of the restrictive law in Nigeria, and the need to protect themselves, they often do not disclose what they do. Participants thought that community members often do not know the difference between AC and PAC, and thus stigmatise anyone they associate with abortion.

You see, the issue is that it is really the same, like I have always said, people find it difficult to differentiate between post abortion care and abortion care. For them, it is the same thing. Whether it is post-abortion care or abortion care, it is the same thing … (Interview 5, medical doctor, private)

We also explored possible gender differences in experiences of stigma. There was a perception among some participants that there is no difference in the way male and female AC and PAC providers are treated. However, another view was that male providers are more confident and resilient in the face of stigma than their female counterparts.

No, there is not much difference. Just that the male PAC providers, they have much more confidence than we, they have much more confidence than we the female PAC providers. (Interview 8, nurse, public)

There was some speculation that this may be because male providers outnumber female providers.

However, a different perspective was that female AC providers are more stigmatized than male providers because women are regarded as a link to life and should be seen preserving and not terminating a pregnancy. Such a view resonates with a constituent element of abortion stigma as representing a rejection of maternalism.

Yes, there is, because they don’t expect that as a female you should be dabbling into that area, because you are supposed to be like a link toward life. For men, they can easily be forgiven. When males are stigmatized, they tend not to care much. They don’t care as much as the females. (Interview 2, nurse/midwife, private)

Our analysis also points to the perceived severity of stigma increasing with pregnancy duration, with abortion care in the first trimester being considered somewhat acceptable. Second and third trimesters are associated with higher levels of stigma.

Yes, the person who does an abortion in the third trimester will be seen as being very wicked. The question is why would you allow it to get to the third trimester before terminating the pregnancy? But the first trimester may be acceptable provided there is a good reason for it. But by the second and third trimesters, society will frown at that. I know of somebody who told me how wicked a doctor was when he brought out a baby and the baby was still breathing, and he had to suffocate the baby to die. That was not good and people were really saying bad things about that. So, I think the first trimester is acceptable. By the time, more the months pile up, the more it becomes unacceptable. (Interview 3, medical doctor, public)

More positively, we identified views that abortion stigma is reducing, particularly from colleagues in the medical profession.

As at ten years ago, there was segregation, there is a kind of other health workers that don’t provide such health care services whereby, a patient goes to them to receive such services, they will shout at them and ask them to go to another place. Even at meetings of doctors (e.g., the Nigerian Medical Association), some of them will jeer at others by saying ‘your people, how many have you done today?’. However, these days, this is no longer frequent, because everybody knows that all we are trying to achieve is to reduce, child and maternal death rates. (Interview 7, medical doctor, private)

### Impact of abortion stigma experiences

We have already touched upon the impact of abortion stigma experiences on professional and personal lives. This theme is developed in this section where we analyse the extent to which the participants internalize stigma through self-blame and feeling that they are doing something wrong. We also consider how experiencing abortion stigma may negatively affect professional practice, through feelings of professional and social insecurity resulting in a tendency for them to practice induced abortion secretly.

Participants spoke of internalising stigma, typically through feeling guilty and despondent. Uncomfortable comments from people in the workplace and community are a common experience, whereby some have got used to hearing it and it has no effect, while others are careful not to be found where such words will be said, or still feel guilty, rejected and condemned because of stigmatization of their work. The feeling of guilt emanating from judgemental statements and attitudes of fellow providers and others was described as emotionally draining.

For me sometimes I feel emotionally exhausted, I also feel guilty sometimes, because of the kind of cases you would have, like I mentioned, second trimesters, sometimes you feel guilty, and some people will quote for you that the bible says we should not kill, even if someone is pregnant and that the bible says that we should multiply on the earth, so getting involved in abortion, you are going against God’s precept and law, sometimes your mind will also, and then you feel guilt at the end of it. (Interview 9, nurse, NGO)

Some AC and PAC providers spoke of a view among colleagues both within and external to abortion services, which associates abortion work with negative life events such as prolonged involuntary childlessness. One of the interviewees narrated the example of her colleagues.

I have some colleagues who have not had any children, most of them have stayed like five, or six years in marriage and they have no children. Sometimes, they feel that the abortion services they render have a hand in their childlessness. Some colleagues outside also link it to possibly what they are doing because they are removing other peoples’ children doing abortion, that maybe the cause of their not being able to give birth. (Interview 9, nurse, NGO)

Many of the narratives expressed insecurity, which involved having to be careful in providing the service so they are not arrested or harassed, not only by law enforcement agents but also by relatives of the women. They ensure that recommended procedures such as completing a consent form and writing an acceptable indication are followed. Sometimes, there is a feeling of despondency, amidst the conviction that life needs to be saved.

Being rejected and isolated affects my social relationships, I feel isolated and rejected. I sometimes feel guilty within me. This is the work I have been doing since 2017 to save lives; doing this kind of job which is not acceptable to the community, you don’t have a choice. (Interview 8, nurse, public)

The providers also described feeling unprotected and insecure. This affected their freedom of association and expression in social circles and even before law enforcement agents. They withdraw from forums or places where the issue of abortion is discussed or say nothing if they have to be there.

With something like this, you don’t want to flaunt yourself in anything. For example, when they wanted to make me a Deacon in the Church (Church leader), I told them I was not interested. Later, because I did not want them to think I was hiding something, I accepted to be an elder. What I am trying to say is, when you know you are giving these services, you cannot feel free to express your opinion on many issues. You have to settle that quietly and let it be and, in any circle, you bring it up, they will view you in a cynical way, that is why we tend to be silent on many aspects. (Interview 6, medical doctor, private)

As illustrated by this medical doctor, often providers give partial or no information about their work, to avoid being judged and stigmatized outside the workplace. Participants also indicated that the secrecy and insincerity they felt obliged to practice did have a negative impact on their clinical practice. To shield themselves from stigma and law enforcement agents’ harassment, AC and PAC providers reported not discussing their work fully. This involves a world of secrecy and silence, key outcomes of abortion stigma, with implications for care.

It is actually not nice … you know medicine is not a profession where lies are permitted, you know you are a medical doctor [and] you know that in a medical exam if you lie or say what is not there, you fail that exam immediately, you know in an oral exam you don’t just say what you didn’t see or what is not there. These things are making us spoil our ethics and making us do things that shouldn’t be done. I feel bad when I see all the shortcuts and all the unethical things going on just to cover tracks on abortion care and all that, … there is a lot of secrecy because of all this stigmatization the provider is secretive, and the client is secretive. (Interview 3, medical doctor, public)

As well as speaking of their own experiences, participants spoke of the effect of abortion stigma on others. They felt that health care professionals are discouraged from providing AC and PAC because of the stigma associated with the work

There are lots of [health] professionals who would have loved to provide these services, but they stay away, because of all these side talks, it is not easy really, you have to be a strong person to move around and pretend that it doesn’t matter, it is not everyone who can do that. … (Interview 5, medical doctor, private)

There were incidents recounted of the providers being humiliated out of their service location.

This is a Muslim environment we work in. I have noticed that many Muslims, those who provide abortion care, do so with a lot of discrimination. I know a friend of mine … [working in] clinic and maternity who was sent packing from his former location because he was offering abortion services. So many of the people [providers] around here especially in the Muslim environment have had to move, and move, because of the fact that they provide abortion care. Even though I have not had any personal incidences like that, I have seen that many abortion providers have been stigmatized and forced to leave their place of work. (Interview 3, medical doctor, public)

### Stigma resistance and conscientious commitment

We have outlined how stigma makes providers feel condemned, rejected, and isolated. However, we can also identify conscientious commitment. This stems from a feeling of satisfaction that they are saving lives.

I know within me that I am saving a life. I am saving the life of others by carrying out this job … I am saving a life, I am helping to prevent unwanted pregnancy, so there is nothing I can do. Like, for a patient that comes for family planning it is normal for me to do pregnancy test for the patient, and by doing this I find out that the patient is positive, so the next thing is if the patient doesn’t want it, you can, either do the clinical or medical abortion, and go on with the family planning. (Interview 8, nurse, public)

The commitment exacts a high price, as the next participant makes clear.

Because I apply the uniform, I try not to think too badly because I am paid to do the job, but within me, when I actually leave that place, I do feel like people, I don’t know what people, are thinking about me, that I am. that I am a murderer or something. That is the way I feel … I feel bad, I feel bad, but saving life is uttermost. I love the work I do; I save lives, and I try to make people have their life and their future back. So, I am happy doing the work I do. (Interview 2, nurse/midwife, private)

This extract captures the negativity of stigma but also resistance. Despite feelings of stigmatization, the respondents also reported a sense of ease in their role in providing abortion care, as well as resilience. This was largely grounded in an understanding that services are a medical necessity; they are providing quality and skilled care minimizing the risks associated with women accessing informal services or taking their own lives. As such, they draw upon the knowledge, that they are potentially saving a life (as in the quotation above) to help counter the stigmatization that could come; and a number of other resistance narratives.

Like I mentioned earlier, my joy at the end of the day is that I am saving lives, I am saving lives, seriously I am saving lives. … so that is just my joy that at the end of the day I am saving lives and not just one life, many lives. Just to help them understand that they also have a chance, to help them have a better future for themselves in as much as they are able to take care of the situation they are at the moment … it doesn’t hinder them to have a brighter future … My joy at the end, not just the salary or whatever they are paying, my joy is that at the end of the day I am saving lives … Thank God I was able to interfere and help. … I will tell myself thank you to God because you made me save a life today from committing suicide. Because most of them have tried suicidal acts severally, but then I was able to intercede and save a life, that is what I would say at the end of it and I am happy. I am happy that no matter what. (Interview 9, nurse, NGO)

As well as talking very seriously about saving lives, this nurse also talks of enabling patients to have a ‘better future for themselves’. This was an important theme in the research:

I am okay, I feel, every day I solve people’s problems … I saw this and felt that I could be helpful. I could help these young ladies realize their dreams. (Interview 5, medical doctor, private)

For others, there appears to be a quiet satisfaction in doing an important job, and simply not being too troubled by the stigma:

Actually, I am very comfortable with the work I do, wherever I am practicing, I am viewed as someone who is providing essential and safe services. (Interview 4, medical doctor, public)

I know that every patient I work with appreciates my service and they thank me for it, so the patient appreciates what I do, and I have good reasons to do what I do, so there is … no, I don’t feel anything and I don’t depend on peoples opinion. (Interview 3, medical doctor, public)

## Discussion

The objective of the study was to investigate the experiences of stigma among providers of safe abortion care in Nigeria. To the best of our knowledge, this is one of the few studies that have explored health providers’ experiences of stigma as a social consequence of the provision of safe abortion care in the country. Given the widely reported cases of stigma in other parts of the world [21–23], the highly restrictive abortion law in Nigeria [2], and the prevailing religious, cultural, and ethical debate surrounding its practice [16–18], we hypothesized that stigma would feature as a major social consequence which would limit the delivery? of safe abortion care in the country. The results of this qualitative study confirm the perception and experience of stigma by the health professionals interviewed. They are also suggestive of the systemic underpinnings of stigma as a product of broad social, cultural, and community contexts.

Two categories of safe abortion care were considered in this study: abortion care and post-abortion care. While AC denotes the safe termination of an unwanted pregnancy, PAC relates to the treatment of complications arising from a botched induced abortion or a spontaneous abortion as defined by the WHO [24]. The results of this study suggest that AC providers may suffer more social stigma than providers of PAC. While PAC providers can be regarded as ‘lifesavers’, providers of AC are often labelled as ‘killers’. This differential connotation of AC and PAC providers has previously been reported in studies in Burkina Faso [25], Kenya [26], and Ghana [27], and was suggested as likely due to limited community awareness about the legal framework for abortion such as life saving and post-abortion care and the requirement for safety in carrying out the procedures. It suggests the need for interventions that address misinformation about abortion and safe abortion care and general awareness of the law and legal status of AC and PAC in various population groups in the country.

Participants reported various dimensions of abortion stigma, including disapproval and disrespect towards providers, name-calling, labelling, and societal judgement and social exclusion. Facilities were often tagged and profiled by anti-abortionists. Stigma led to several consequences of stigma, such as fear for their safety, direct attacks by relatives, fear of police harassment, clandestine practices by providers, despondency, and emotional exhaustion. Additionally, providers faced other stigmatizing consequences associated with providing abortion care including unfriendly attitudes from both professional and non-professional colleagues, a lack of professional incentives, fear of job loss, and negative impacts on their private life.

This pattern of experiences of abortion stigma has also been reported in other sub-Saharan African countries where abortion is legally restricted. In a systematic review of published articles, Zia et al. reported high rates of stigma, shame, and abandonment associated with abortion and post-abortion care in several African countries [28]. The paper concluded by recommending several coping mechanisms to deal with stigma among health professionals providing safe abortion care.

Although physical violence targeting abortion providers occurs in several settings around the world [29], this was not specifically reported in this study. Similarly, although the restrictive abortion law in Nigeria makes provision for jail terms for providers, there have been no known instances of successful legal prosecution of providers in the country. No jail terms or successful prosecutions of providers were reported by participants in this study. Nevertheless, the participants in this study pinpointed the fear of police and legal actions as additional stigmatizing outcomes of abortion care, which prevents the regular provision of safe abortion care by health providers. These are very real structural features of abortion stigma.

We investigated the notion of alternative reasons for abortion stigma by asking participants about their perception of the cause of abortion stigma in Nigeria. The results showed the perception of abortion stigma being attributable to cultural and religious reasons. There was also a consensus among the respondents that abortion stigma is attributable to a form of hypocrisy whereby those who themselves practice abortion in secret turn around to denounce the practice publicly. Cultural and religious factors have featured over time in debates about abortion care in Nigeria. Most major religions in the country condemn the practice of abortion, but our previous studies have shown that adherents of all religious groups have been known to use abortion care [30-33], confirming hypocrisy as a reasonable thesis that illustrates some abortion prevalence but in secret for those that are able to maintain silence. While the promotion of open debate about abortion and the human rights prerogative of women to decide on abortion may limit this kind of hypocrisy, it is possible that the movement towards decriminalization of the abortion law as has happened in neighbouring countries such as the Republic of Benin, the Central African Republic [34], and Ghana [35,36] may be the most urgent solution to the problem.

The results of this study indicate that the fear of stigma and public disapproval contributes to health practitioners’ reluctance to provide abortion care, limiting women’s access to safe abortion and post-abortion care in the country. This finding is consistent with reports in the literature which highlights abortion stigma as a factor in the low practice of safe abortion care in countries with restrictive abortion laws [37]. The results also suggest that some health providers are unaware of the specific provisions of the abortion law, mistakenly believing all abortions are prohibited in the country. Targeted specific interventions to raise awareness of the law and the conditions outlined by the Federal Ministry of Health [3] for terminating unwanted pregnancies could increase providers’ confidence in offering safe abortion without feeling guilt, shame, or stigmatisation.

### Study strengths and limitations

This study has both strengths and limitations. The major strength is its scope, involving interviews with health providers from both the northern and southern regions of Nigeria. As abortion is on the federal and national concurrent list of health decision-making, it is evident that only research and interventions that include participants from various parts of Nigeria are essential for addressing the gaps and challenges in providing safe abortion care.

A further study that includes experiences and notions of stigma by women seeking abortion services, those who oversee abortion and related policies, as well as international organizations that support safe abortion care in Nigeria, would extensively document the nature of abortion stigma in the country more extensively. This study provides relevant research that can spur further studies and policy/programmatic review to enable a proper understanding of the nature of the depth of stigma related to abortion care in Nigeria.

## Conclusion

We conclude that health professionals providing safe abortion services in Nigeria experience and perceive stigma as a result of their work. These are attributable to cultural and religious notions of morality as well as the restrictive abortion law and manifestations in intermediating spaces. These spaces are within the workplace, communities, and personal social networks where stigma can affect healthcare providers’ ability to speak freely and engage effectively. While legal change is important, its impact may be limited without interventions that address stigmatizing attitudes within both the community and the workforce. Efforts to address these inter-mediating factors could reduce the level of stigma and improve women’s access to safe abortion care in the country.

## Data Availability

There is no dataset publically available to ensure the anonymity of participants.
